# Characteristics and Mechanisms of Phosphorous Adsorption by Peanut Shell-Derived Biochar Modified with Magnesium Chloride by Ultrasonic-Assisted Impregnation

**DOI:** 10.1021/acsomega.2c05474

**Published:** 2022-11-14

**Authors:** Xiaoqi Liu, Wei Zhou, Lei Feng, Lulu Wu, Jialong Lv, Wei Du

**Affiliations:** † College of Natural Resources and Environment, Northwest A&F University, Yangling District, Xianyang712100, Shaanxi, China; ‡ College of Resource and Environment, Xinjiang Agricultural Universit, Urumqi830052, China

## Abstract

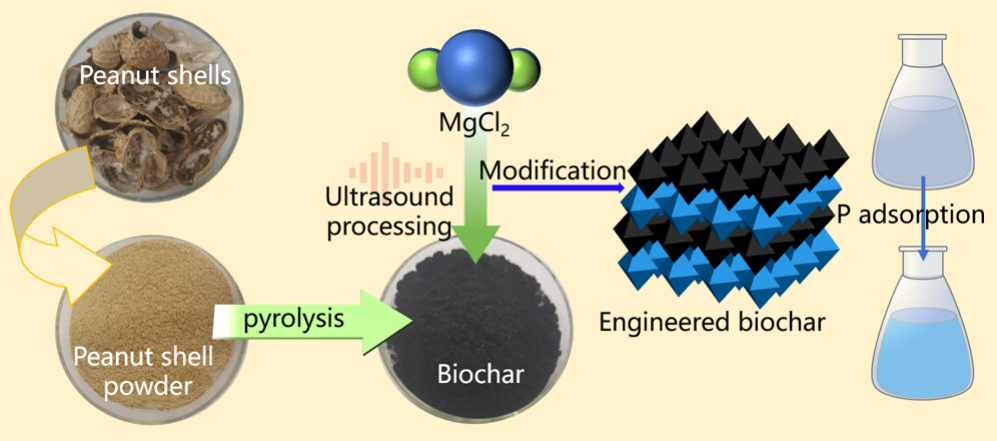

Recovery of phosphate (P) from sludge, domestic wastewater, and industrial wastewater is beneficial for overcoming the problem of shortage of P rock resources. In this study, Mg-functionalized peanut shell-derived biochar was prepared by ultrasound-assisted impregnation. The obtained Mg-laden biochar had a higher content of Mg, a larger specific surface area, and more porosity. The prepared Mg-modified biochar exhibited excellent adsorption properties of phosphorus. Modified biochar has a higher amount of adsorbed P than raw biochar. The capacity of P adsorption by modified biochar was 30.48–114.24% higher than that by raw biochar. Moreover, the Mg-laden biochar can be applied in a wide working environment (pH: 2–10; temperature range: 15–40 °C). This study not only develops a new strategy for the preparation of high-capacity P adsorbents but also provides a new green use for agricultural peanut shells.

## 1 Introduction

Phosphorus (P) is one of the most important essential nutrients for plants as it is involved in many important physiological reactions, such as the synthesis of DNA, RNA, ATP, and phospholipids during plant growth and development.^1−4^ The application of P fertilizers is important for food production because P deficiency is widespread in most soils.^5^ In recent decades, the production and consumption of P fertilizers have increased dramatically due to a steady global population increase, coupled with an improvement in diet structure.^6^ According to statistics, an estimated 263,000 tons of phosphate rock (PR) were mined worldwide in 2017.^7^ The increasing mining of PR and the fact that PR is a nonrenewable resource led to the European Commission declaring PR as a critical raw material in 2014.^8^ The demand for P is increasing globally, despite limited PR resources, due to an increasing population and global trends toward more meat consumption and dairy-based diets which are significantly more P intensive.^9^ It is estimated that the current PR reserves would be fully depleted in around 372 years at the current rate of mining.^10^ The world is facing this shortage of PR resources.

In recent years, scientists, governments, and industry have all gained a new understanding of the recovery of phosphorus from municipal or industrial wastewater, and it is believed that the P recovered from wastewater has the potential to substitute a significant portion of the demand for PR.^11,12^ In fact, many studies have been conducted to recover P from a variety of wastewaters, such as membrane concentrates,^13^ aquaculture wastewater,^14^ swine wastewater,^15^ saline industrial wastewater,^16^ etc. Meanwhile, researchers have developed many approaches to recover P from wastewater, including chemical precipitation, crystallization, ion-exchange processes, membrane processes, electrochemical processes, and biological processes.^17−19^ However, these methods often require high costs of ions and chemicals, have complex operating procedures, and cause massive waste production.^20^

Adsorption is a low-cost and easy-to-operate technology that can help remove and recover P from water.^21,22^ Biochar has been widely used to recover P from wastewater due to its stable structure, rich porosity, large specific surface area, and rich functional groups, such as hydroxyl groups, amine, alkyl, amide, and ether groups.^23−25^ However, raw biochar displays a limited capacity for anionic pollutant removal, including P, which may be due to limited functional groups,^26^ and raw biochar usually presents a high amount of negatively charged groups on their surface (−OH, −COOH), which may be ineffective for the removal of P.^27^ Therefore, it is necessary to modify raw biochar to enhance the adsorption capacity. Jung and Ahn prepared porosity-enhanced biochar containing periclase (MgO) nanocomposites (PE-MgO/biochar) using a novel combined electrochemical modification method.^28^ Takaya et al. reported that biochar P adsorption can be enhanced from relatively low levels (e.g., 2.1–3.6%) to relatively high levels (66.4–70.3%) by impregnation with magnesium.^29^ In recent years, substances used for modifying biochar are mainly MgO, MgCl_2_, La (OH)_3_, LaCl_3_, Na_2_CO_3_, NaOH, and FeCl_3_.^31−33^

The annual output of peanut shells (appendages in peanut production) is about 5.2 million tons in China.^34^ Most of the peanut shells are disposed by incineration or landfill, which causes a waste of resources and a huge impact on the environment. Preparation of biochar from peanut shells is a green, sustainable, low-cost, win–win production method, which can not only reduce the pollution caused by peanut shells but also be used to remove P from wastewater. Ultrasound is a type of energy that can enrich the pores of biochar and increase the functional group content of the surface.^35,36^ In this experiment, peanut shells were used to prepare biochar. In addition, we evaluated the P adsorption properties of Mg-loaded biochar prepared under different ultrasound waves.

## 2 Materials and Methods

### 2.1 Materials

Peanut shells were provided by Shijiazhuang Dasong Agricultural Planting Co. Ltd. (Hebei, China). All chemicals used in the present study were of analytical reagent grade (>99.0% purity).

### 2.2 Biochar Production

Peanut shells were washed with deionized water 3 times, then naturally dried, crushed, and sieved through a 100-mesh sieve. The peanut shell powder was dried at 60° for 24 h. Then, the peanut shell powder was placed in a programmable tube electric furnace and heated up to 550 °C with a heating rate of 15 °C min^–1^ and a holding time of 2 h under a N_2_ atmosphere. Thereby, peanut shell-derived biochar samples were obtained and denoted as PSB. The element contents of C, N, and O are shown in Table S1.

The Mg-laden biochar was prepared as follows: 25 g of PSB was added to 500 mL of 0.5 M MgCl_2_ solution, and the pH was adjusted to around 8.0 with 0.1 M HCl or 0.1 M NaOH. The mixture was incubated with constant stirring at 25 °C for 24 h and filtered; then, the solid was dried at 105 °C and named MPSB. In addition, we evaluated the performance effect of MPSB prepared under different ultrasound interventions. In brief, PSB was pretreated in an ultrasound environment before incubating the mixture of MgCl_2_ solution and PSB. According to the different ultrasound healing times (10, 20, and 30 min), three samples were obtained and named UMPSB1, UMPSB2, and UMPSB3, respectively.

### 2.3 P adsorption

P solutions were prepared by dissolving monopotassium phosphate (KH_2_PO_4_) in deionized water. The detailed steps of the P adsorption experiment of the sorbent were as follow: (1) 0.05 g of the sorbent was added to 50 mL of phosphate solution with 10–200 mg L^–1^ P in 100 mL containers, respectively. (2) The containers were then shaken using a shaker with a rate of 180 rpm at 25 ± 0.5 °C for 24 h. (3) The mixtures were filtered through a 0.22 μm filter membrane, and the concentration of P in the filtrate was determined using ammonium molybdate and ascorbic acid.

The P adsorption kinetics of the adsorbent were addressed by adding 0.05 g of adsorbent into 50 mL of phosphate solution (200 mg L^–1^ P) in 100 mL containers. Then, these containers were shaken using a shaker with a rate of 180 rpm at 25 ± 0.5 °C for 0.5–24 h. Afterward, the mixtures were filtered, and the P concentration in the filtrate was determined. The P adsorption capacity of biochar could be characterized according to the P concentration difference in solution before and after adsorption experiments.

The adsorption capacity of the adsorbent was calculated using the following formula
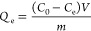
1where *Q*_e_ is the amount of adsorption (mg P g^–1^), *C*_0_, *C*_e_, *V*, and *m* are the initial *P* concentration (mg L^–1^ P), equilibrium solution (mg L^–1^ P), the volume of solution (L), and the weight of adsorbent (m), respectively.

The Langmuir equations (eq 2) were employed to evaluate the adsorption, and second-order mathematical models (eq 3) were used to simulate the adsorption kinetics
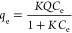
2
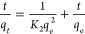
3*Q* is the Langmuir maximum capacity (mg g^–1^), *C*_e_ is the equilibrium solution concentration (mg L^–1^), and *K* and *K*_2_ represent the Langmuir bonding terms related to interaction energies and constants, respectively. *q_t_* and *q*_e_ are the adsorption capacity at time *t* and at equilibrium, respectively (mg g^–1^).

To investigate the effect of ambient temperature on the P adsorption performance of the adsorbents, a series of adsorption experiments with different ambient temperatures from 15 to 40 °C were performed by mixing 0.05 g of adsorbent and 100 mL of phosphate solutions (200 mg L^–1^ P).

To explore the effects of initial pH values on the P adsorption capacity of adsorbents, a series of adsorption experiments were performed in phosphate solutions (200 mg L^–1^ P) with different initial pH values from 2 to 12. The initial pH was adjusted with 0.1 M HCl and 0.1 M NaOH solutions.

### 2.4 Adsorbent Regeneration

The recycling ability of adsorbents was explored in this study. The adsorption experiments were carried out by mixing 0.1 g of adsorbents and 200 mL of 200 mg L^–1^. The P-laden biochar samples were then added to 100 mL of NaOH solution (3 M) and shaken for 12 h. The regenerated adsorbents were washed with deionized water and then dried at 80 °C for 12 h. The obtained samples were directly reused in the next adsorption experiment. Finally, the adsorption–regeneration experiments were repeated for five cycles following the same process.

### 2.5 Characterization

Elemental analysis (C, H, N, and S) was performed using a Vario-EL Elemental Analyzer (Vario-EL, Germany). The morphologies of PSB and modified PSBs were investigated by scanning electron microscopy (SEM, QUANTA250). Functional groups of the samples were analyzed by Fourier transform infrared (FTIR) spectroscopy (Nicolet IS10) in the scanning range of 4000–500 cm^–1^. The Brunauer–Emmett–Teller (BET) surface area, total pore volume, and pore diameter of adsorbents were measured using a N_2_ absorption-adsorption spectrometer (BELSORP-MINI II, MICROTRACBEL, Japan). The elemental composition of samples was determined by energy-dispersive X-ray spectroscopy (EDX) and X-ray photoelectron spectroscopy (XPS). X-ray diffraction (XRD) analyses of adsorbents before and after adsorbing phosphate were performed (ADVANCE-D8, BRUKER, Japan).

### 2.6 Statistical Analysis

All experiments were conducted in triplicate, and the average values are reported. Analysis of variance among treatments and mean separation tests (the Duncan’s multiple range test and least significant difference test) were performed using SPSS 22.0. Differences among means and correlation coefficients were considered significant when *p* < 0.05.

## 3 Results and Discussion

### 3.1 Characterization of Mg-Laden Biochar

The physicochemical properties of different biochar samples are presented in Table 1. The loading content of Mg increases significantly with ultrasound intervention. The BET surface area of biochar decreases when Mg is loaded onto biochar but increases in an ultrasonic environment. Similar changes were found in the total pore volume. The average pore volume of biochar after Mg loading became smaller, maybe because low-frequency ultrasound waves can change the apparent structure of matter, such as generating collapses, breaking down pits, and opening microchannels.^37,38^

**Table 1:** Specific Surface Area and Porosity of Different Adsorbents

sample	Mg content (%)	BET surface area (m^2^ g^–1^)	total pore volume (cm^3^ g^–1^)	average pore diameter (nm)
PSB		153	0.24	14.81
MPSB	4.91	133	0.20	11.24
UMPSB1	9.82	173	0.25	14.51
UMPSB2	11.23	171	0.27	12.43
UMPSB3	10.23	166	0.24	13.85

**Figure 1:**
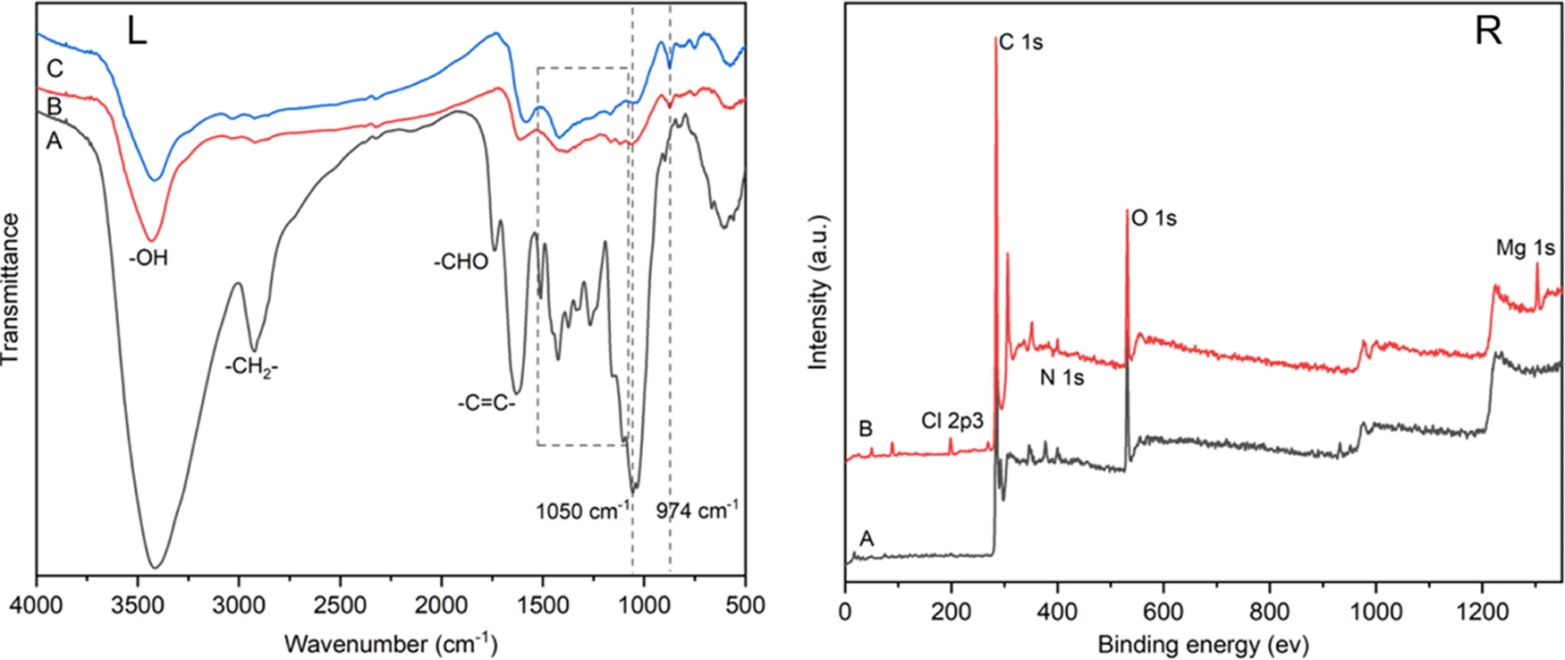
FTIR spectra (L) of PS (L-A), PSB (L-B), and MPSB (L-C); XPS spectra (R) of PSB (R-A) and MPSB (R-B).

**Figure 2:**
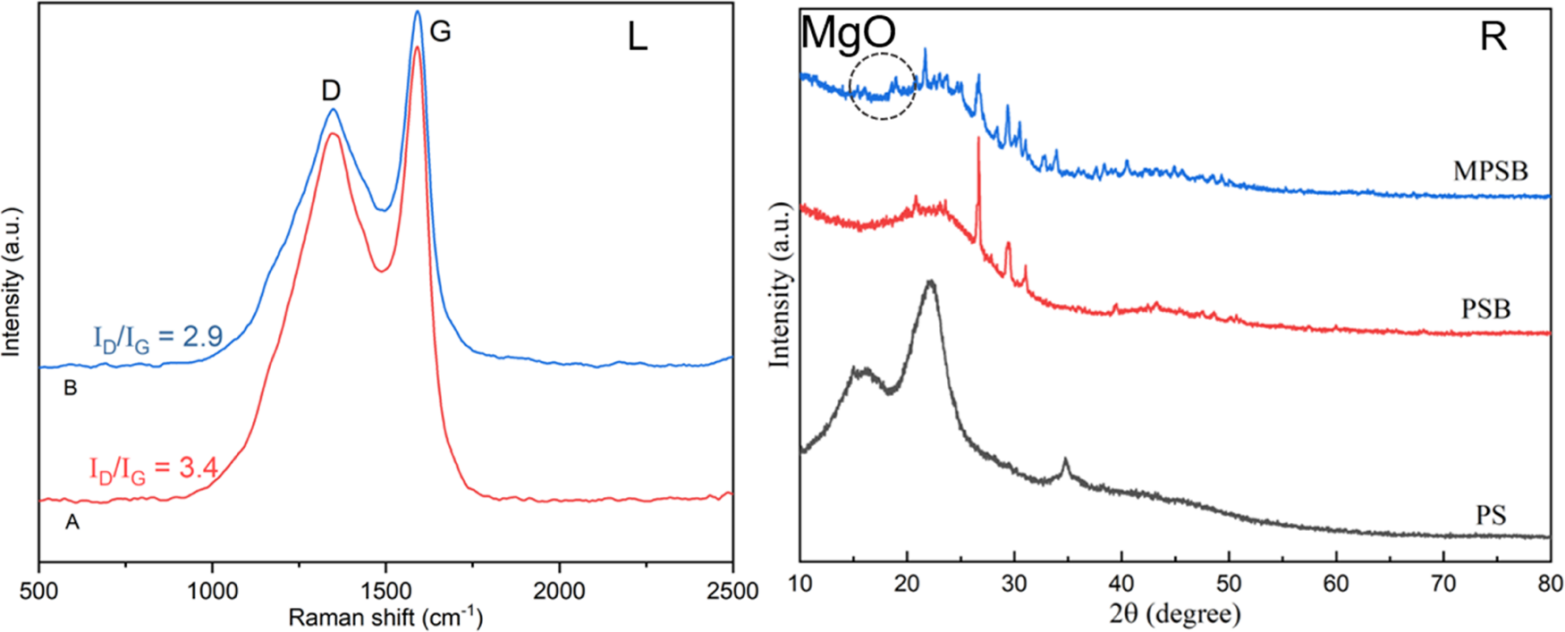
Raman spectra (L) of PSB (L-A) and MPSB (L-B); XRD spectra (R) of PS (R-A), PSB (R-B), and MPSB (R-C).

To confirm the formation of Mg-loaded biochar, FTIR spectra (Figure 1) were obtained to examine the changes in functional groups of different samples. The bands at 3411, 2926, 1740, and 1619 cm^–1^ were associated with the −OH, −CH_2_, −CHO, and C=C groups, respectively. The disappearance of the peak at 1050 cm^–1^ indicated the rupture of the ether bond during pyrolysis. In addition, the peak at about 576 cm^–1^ was assigned to the Mg–O composite (Figure 1L-C).^39^ This was consistent with the results of XRD spectra (Figure 2R). The peak of the Mg–O band could be observed on the MPSB. This was similar to the results of the study by Jiang et al.^40^ The XPS spectra of PSB and MPSB also confirmed that Mg was successfully loaded onto biochar (Figure 1R). As shown in Figure 1R-A, O 1s (531.21 eV), N 1s (399.97 eV), and C 1s (284.44 eV) peaks were observed on the XPS spectrum of PSB. Compared with the XPS spectrum of PSB, Mg 1s (1304.17 eV) and Cl 2p3 (198.79 eV) peaks were observed for MPSB (Figure 1R-B), which denote the existence of Mg. The Raman spectra of PSB and MPSB (Figure 2L) show two fundamental vibrations at 1350 and 1580 cm^–1^, which can be assigned to the D and G bands, respectively. The higher *I*_D_/*I*_G_ intensity (2.9 and 3.4) suggests that the prepared samples have high activity.

**Figure 3:**
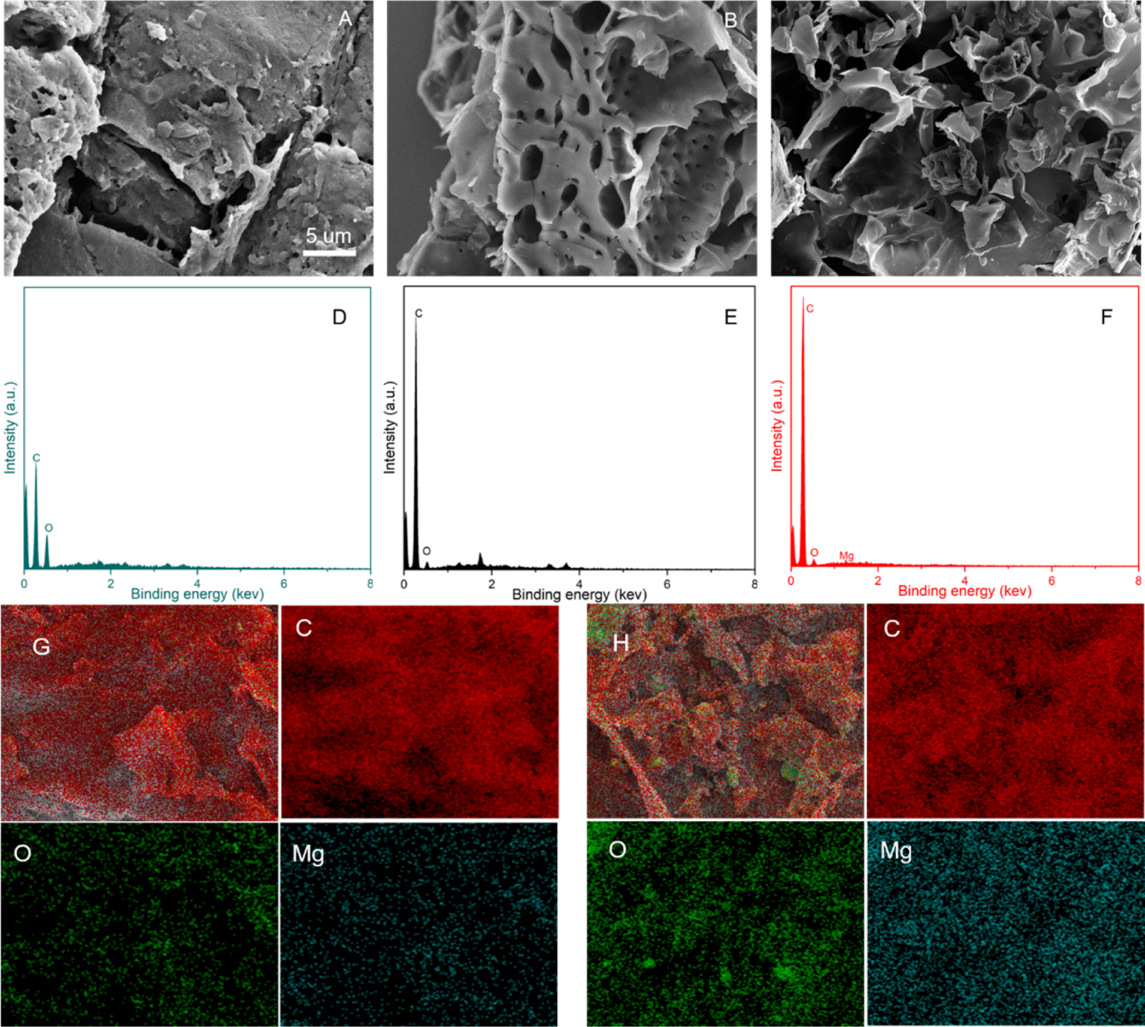
SEM images of PS (A), PSB (B), and MPSB (C); EDX spectra of PS (D), PSB (E), and MPSB (F); EDX maps of PSB (G) and MPSB (H).

To further understand the surfaces characteristics of biochar samples, SEM was employed to examine the surface morphology (Figure 3). There were many small holes on PSB after peanut shell powders were pyrolyzed (Figure 3A,B). After modification by MgCl_2_ in an ultrasonic environment, the surface structure of the biochar became looser (Figure 3C), which was probably beneficial for improving the P adsorption performance.^41^ EDX spectra indicated that the surface of the Mg-laden biochar mainly contained C, O, and Mg elements (Figure 3F). EDX mapping (Figure 3G,H) was also employed to study the elemental compositions and distributions.

### 3.2 Adsorption of P onto Biochar Samples

**Figure 4:**
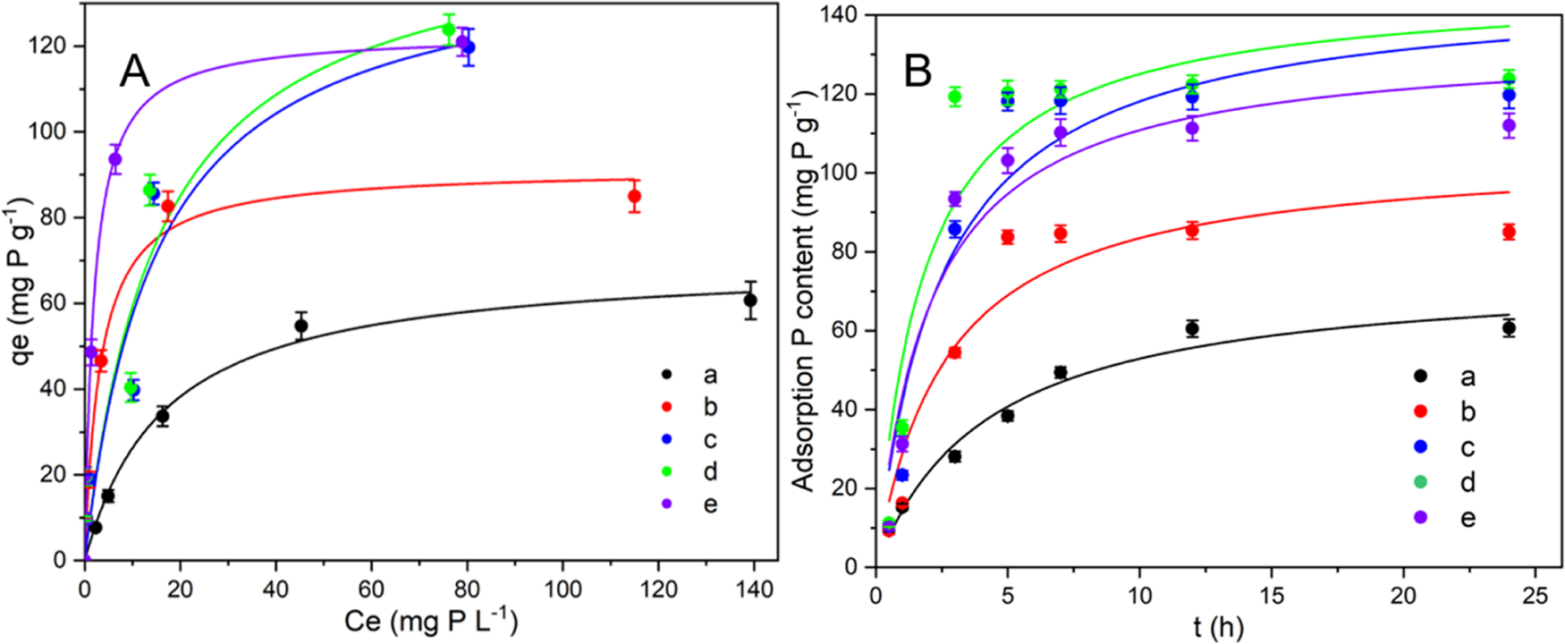
Adsorption isotherm data and modeling for P on different biochars (A); adsorption kinetic data and modeling for P on different biochars (B). a, b, c, d, and e represent PSB, MPSB, MPSB1, MPSB2, and MPSB3, respectively (initial phosphate concentration: 10–200 mg L^–1^ P; adsorbent dosage: 0.05 g; contact time: 24 h).

There are many differences in the P adsorption performance of different adsorbents (Figure 4). All of the five characteristic curves of P adsorption listed in the figure show an inverted “L” shape. However, the adsorption of P by the Mg-modified biochar is greatly improved. In addition, ultrasound intervention also significantly promotes the P adsorption performance of adsorbents. The maximum amounts of P adsorbed by PSB, MPSB, UMPSB1, UMPSB2, and UMPSB3 are 70.09, 91.45, 143.13, 150.16, and 122.98 mg g^–1^, respectively, according to the Langmuir fittings (Table 2), which were much higher than most of the reported adsorbents.^42^ Furthermore, Freundlich and Temkin models for phosphate adsorption onto the biochar were also investigated in this study, and the results are presented in Figure S1.

**Table 2:** Adsorption Isotherm Parameters of Different Adsorbents under the Langmuir Fittings

treatment	*Q*_e_(mg g^–1^)	*K*(l mg^–1^)	*R*^2^	MBC (l g^–1^)
PSB	70.09	6.02 × 10^–2^	0.99	4.22
MPSB	91.45	0.31	0.99	28.00
UMPSB1	143.13	6.7 × 10^–2^	0.92	9.60
UMPSB2	150.16	6.5 × 10^–2^	0.90	9.82
UMPSB3	122.98	0.52	0.98	63.36

To further understand the P adsorption process of different adsorbents, the kinetics of the adsorption process was determined. As shown in Figure 4B, the adsorption of P by absorbents was very fast (∼3–12 h). Second-order kinetic models well described the data of P adsorption (*R*^2^ > 0.9) (Figure 4B and Table 3). The P adsorption process can be divided into two stages: first fast and then slow. In addition, the P adsorption rate of Mg-laden biochar is higher than that of raw biochar. This is consistent with the findings of Xu et al.^43^

**Table 3:** Adsorption Kinetic Parameters of Different Adsorbents under the Pseudo-Second-Order Fittings

treatment	*R*^2^	*k*	*Q_e_*(mg g^–1^)
PSB	0.98	3.14 × 10^–3^	75.32
MPSB	0.91	3.56 × 10^–3^	105.67
UMPSB1	0.91	2.27 × 10^–3^	147.54
UMPSB2	0.87	3.8 × 10^–3^	147.35
UMPSB3	0.92	3.63 × 10^–3^	133.65

### 3.3 Effects of Ambient Temperature and Initial pH Values on P Adsorption

Effects of different initial pH values on P adsorption were also investigated. The response of different adsorbents to pH was basically similar. When the pH of the system was 2–8, the P adsorption capacity of the adsorbent changed slightly with the pH (Figure S2A). This may be related to the strong obligate adsorption of P by the adsorbent in a neutral or acidic solution.^44^ The P adsorption capacity of the adsorbent decreased obviously when the pH increased to 10 (Figure 2A). This result is similar to that of Jiao et al.^45^ Overall, the adsorbent can work in a wide pH range, but the P adsorption capacity was stronger when the pH was less than 10.

Effects of ambient temperature on P adsorption were investigated (Figure S2B). The P adsorption capacity increased slightly with an increase in the ambient temperature from 15 to 40 °C; however, no obvious difference was observed. These results indicate that these adsorbents show excellent P adsorption capacity at room temperature.

### 3.4 Recovery of Phosphate

**Figure 5:**
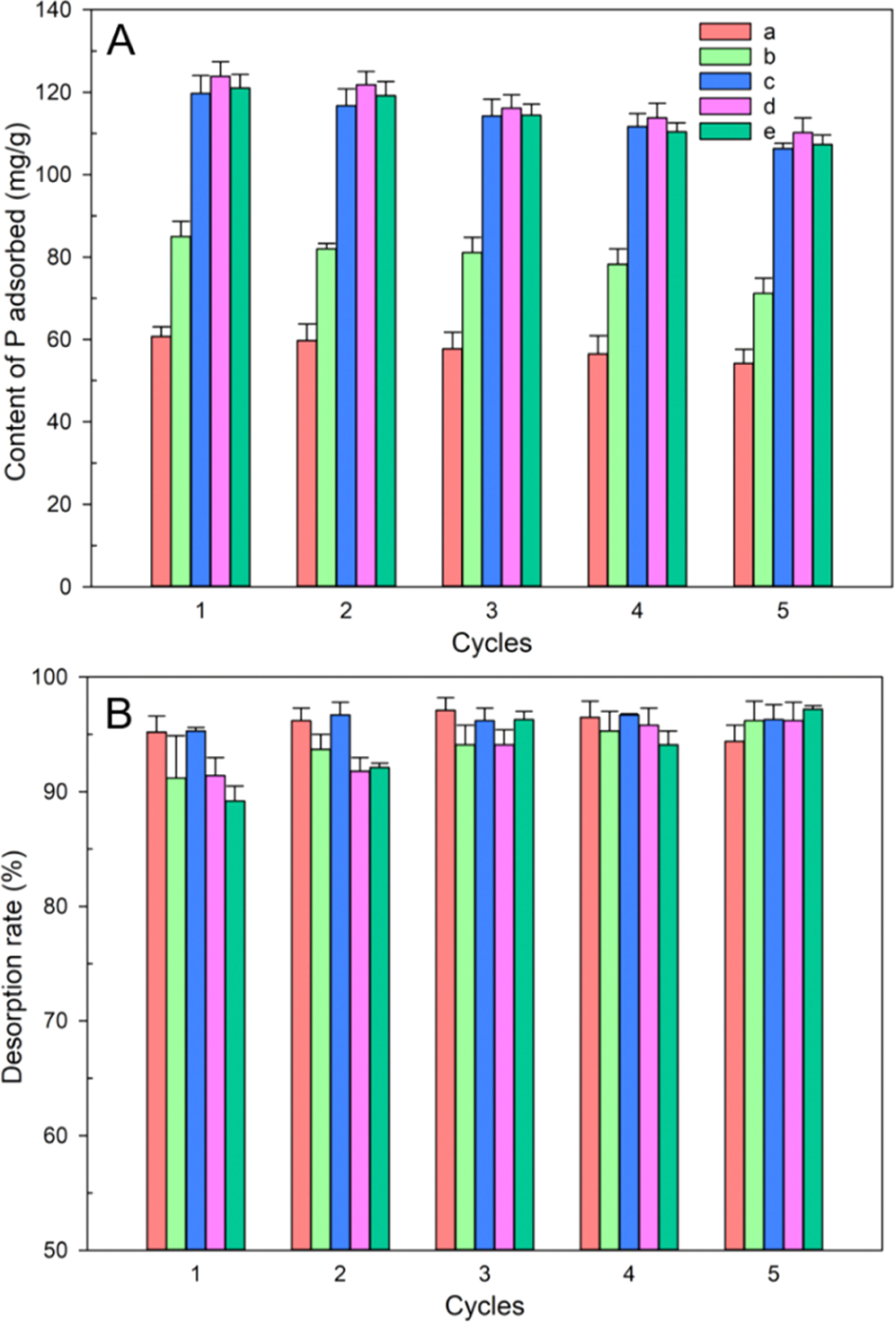
Cyclic adsorption (A) and desorption (B) of phosphorus by adsorbents (NaOH concentration: 3 M; liquid/solid ratio: 100 mL g^–1^; desorption time: 24 h).

The recyclability of adsorbents is an important index to evaluate the properties of adsorbents. With increasing number of adsorbent recycling, the ability to adsorb phosphorus shows a downward trend (Figure 5). However, the adsorbent still shows good adsorption capacity of P after five cycles of adsorption (Figure 5). In addition, the P removal efficiency of each P-laden adsorbent could reach more than 89.2%. This phenomenon showed that the prepared adsorbent had a good P recovery capacity.

### 3.5 Mechanism of P Adsorption

**Figure 6:**
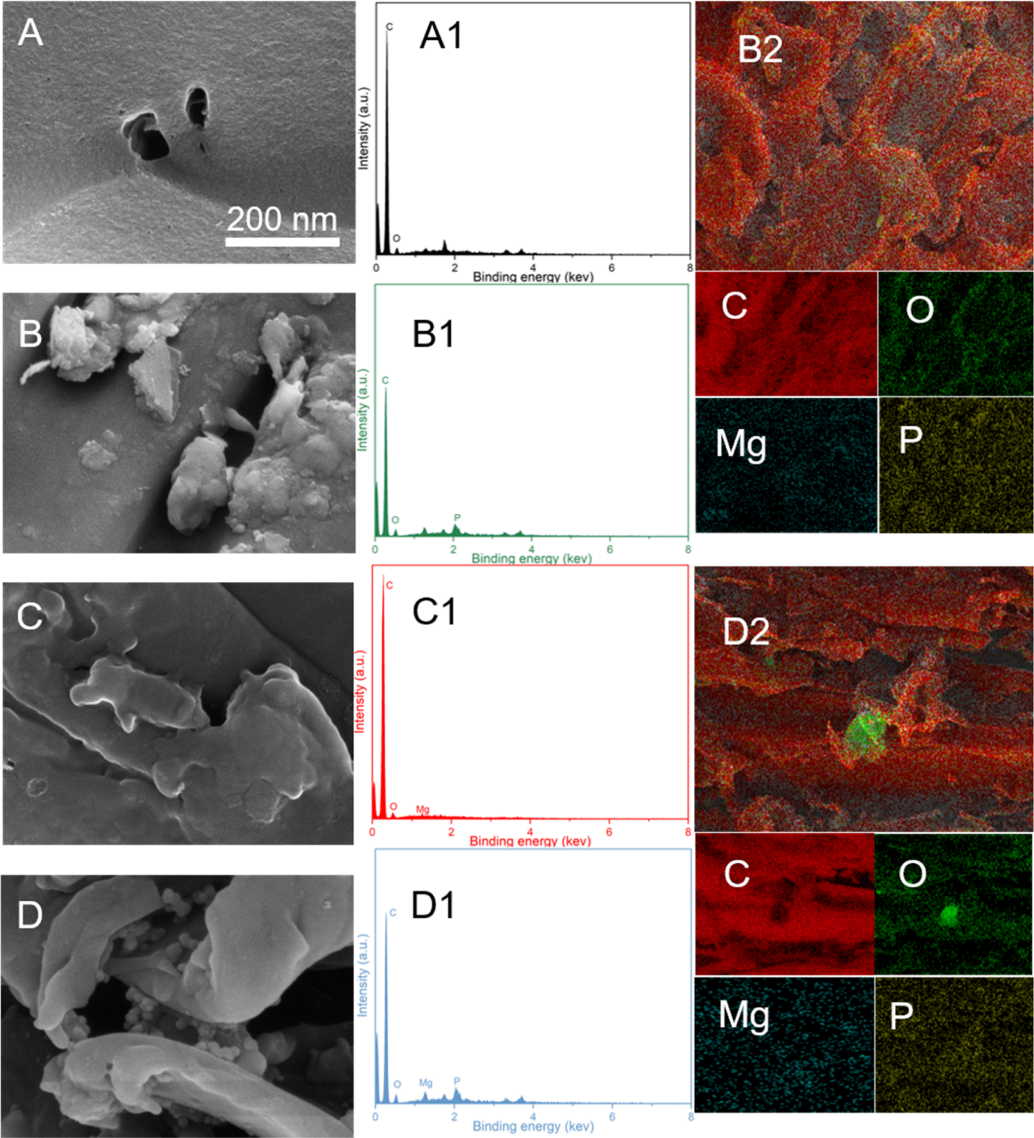
SEM images of PSB (A), PSB after P adsorption (B), MPSB (C), and MPSB after P adsorption (D); EDX maps of PSB (A), PSB after P adsorption (B), MPSB (C), and MPSB after P adsorption (D); EDX maps of PSB after P adsorption (B2) and MPSB after P adsorption (D2).

To elucidate the adsorption mechanism, the surface microstructures of the adsorbent before and after P adsorption were observed (Figure 6A–D). Flower-shaped crystal substances appeared on the surface of adsorbents after P adsorption (Figure 6B,D). The EDX spectra and corresponding mapping confirmed that the flower-shaped crystal substances mainly consisted of O, Mg, and P elements.

**Figure 7:**
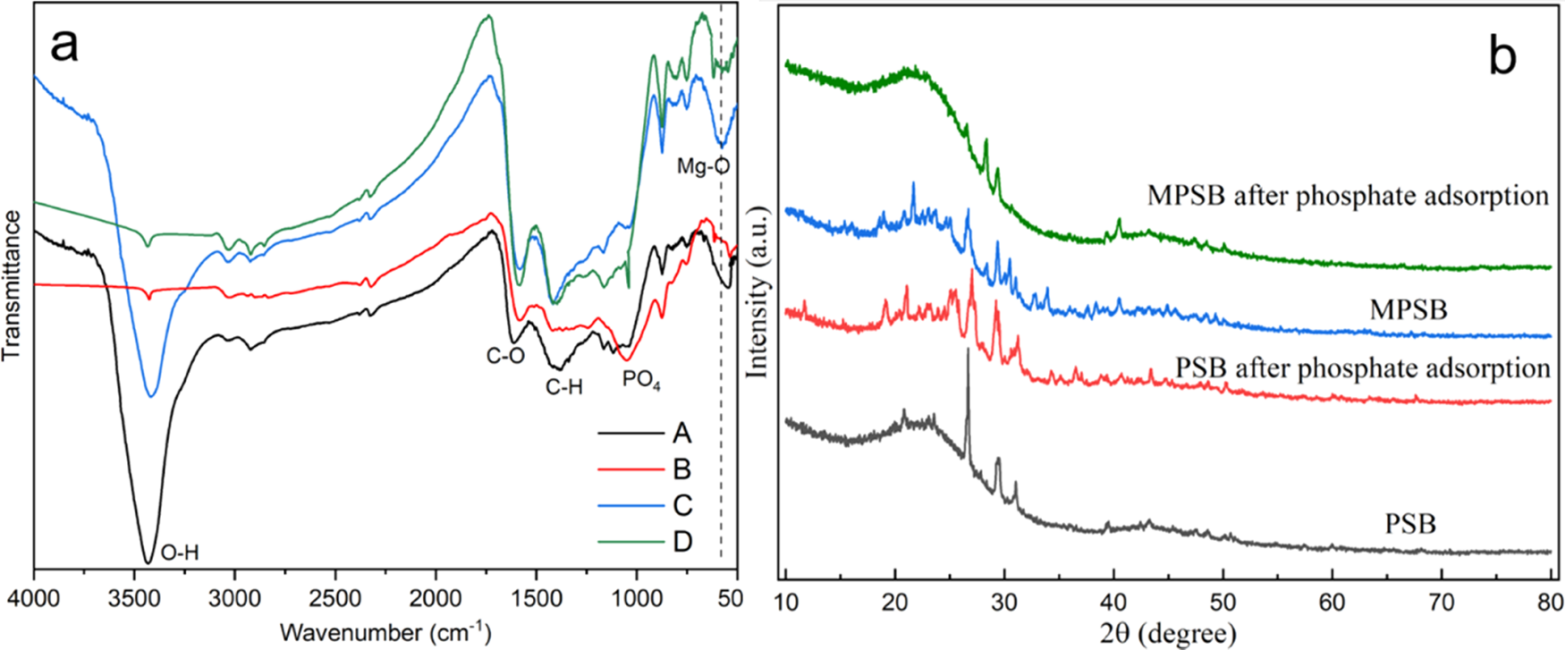
FTIR spectra (a) of PSB (a-A), PSB after P adsorption (a-B), MPSB (a-C), and MPSB after P adsorption (a-D); XRD spectra (b) of PSB, PSB after P adsorption, MPSB, and MPSB after P adsorption.

In addition, the peak at 3411 cm^–1^ weakened significantly after the adsorbent adsorbed P (Figure 7a), which indicated that ligand exchange may occur between the PO_4_^3–^ and −OH.^46^ The peak at about 1050 cm^–1^ was attributed to the PO_4_^3–^ group and the peaks at 620 cm^–1^ (v (O–P)) appeared after adsorption of P, suggesting that phosphate was successfully adsorbed on raw and modified biochar.^47^ The XRD spectra (Figure 7b) also confirm that Mg was involved in the reaction with phosphate.

**Figure 8:**
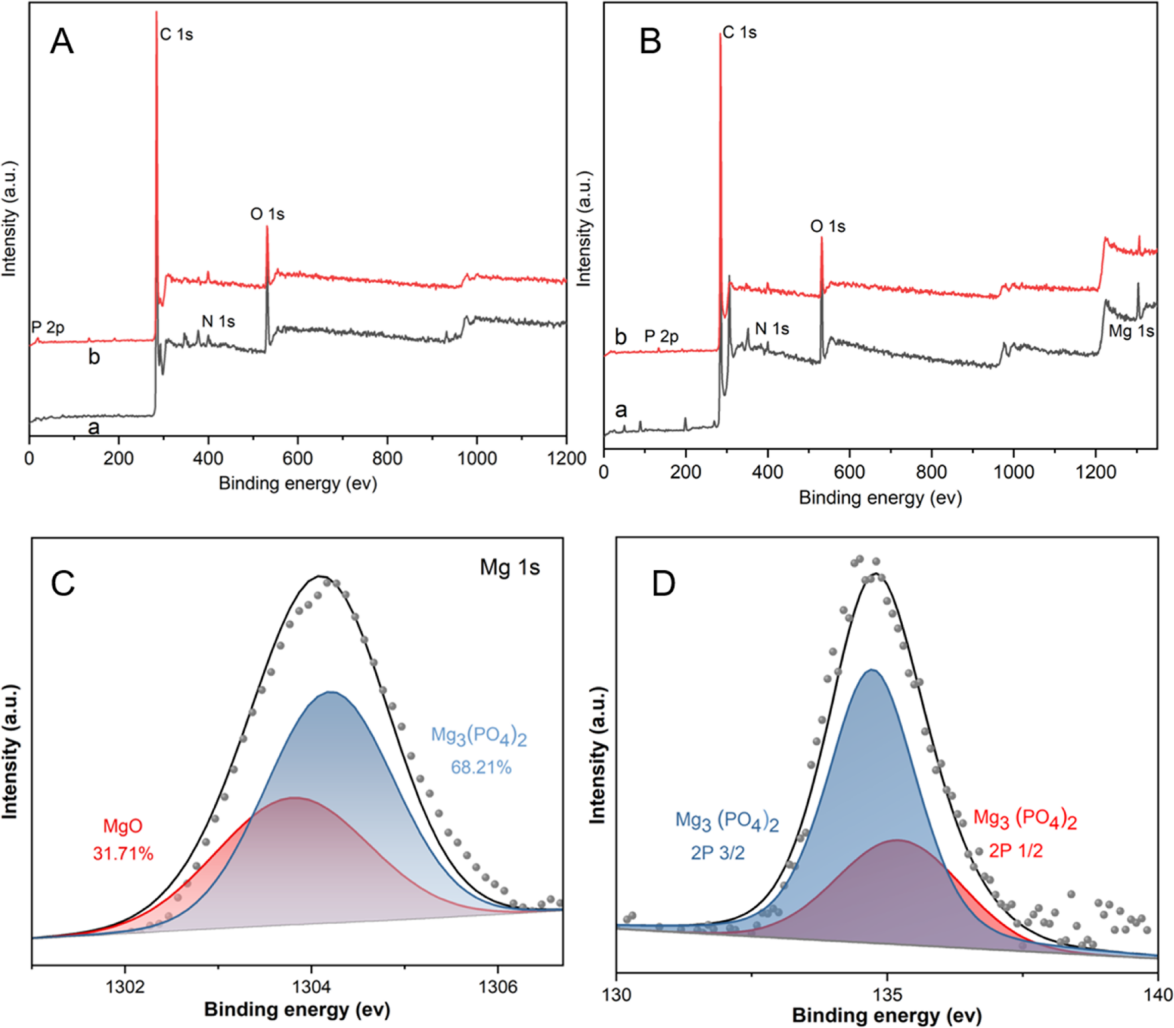
XPS survey spectra of PSB (A) and MPSB (B) before (a) and after (b) P adsorption; M 1s (C) and P 2p (D) spectra of MPSB after P adsorption.

To further investigate the adsorption mechanism, XPS analysis (Figure 8) was performed to determine the elemental compositions and corresponding elemental valence states on the surface of raw and modified biochar before and after the adsorption of P. As shown in Figure 7A-a, O 1s (531.21 eV), N 1s (399.97 eV), and C 1s (284.44 eV) peaks in the XPS spectrum of PSB before P adsorption were observed. However, the P 2p (134.9 eV) peak was observed in the PSB after P adsorption (Figure 8A-b). Furthermore, the signal of Mg 1s was shifted from 1304.17 to 1304.82 eV after P adsorption. The peak of Mg 1s was split into two overlapping peaks, corresponding to MgO and Mg_3_(PO4)_2_, respectively.^48^ The results indicated the Mg_3_(PO_4_)_2_ was formed during phosphate adsorption.

In addition, the effects of the initial pH values on P adsorption showed that there was strong obligatory adsorption during the P adsorption process (Figure S1A), and strong competitive adsorption of OH– existed in the system when the pH was higher than 10. According to the study of Jiao et al., abundant OH– may be generated during phosphate adsorption.^45^

## 4 Conclusions

In this study, we prepared Mg-laden biochar by ultrasonic-assisted impregnation in a magnesium chloride environment. Mg-laden biochar adsorbents prepared under different ultrasonic healing times (10, 20, and 30 min) were evaluated for their adsorption performance of phosphate. Low-frequency ultrasound can increase the content of Mg loading on biochar, specific surface area, and total porosity. The novel preparation method contributes to the formation of the skeleton with more abundant pores. The prepared Mg-laden biochar exhibited excellent adsorption properties in a wide operating pH range (2–10). In addition, the Mg-laden biochar could be regenerated for at least five cycles, and each cycle showed good phosphate adsorption capacity. In summary, we believe the Mg-laden biochar is an excellent adsorbent for removing and recovering phosphate from wastewater.

## Supporting Information Available

C, N, and O element contents of biochar; other models for phosphate adsorption onto the biochar; performance of phosphate adsorption in different environments (PDF)

## Abbreviations

PSB: peanut shell-based biochar
MPSB: Mg-laden peanut shell-based biochar
UMPSB1: Mg-laden peanut shell-based biochar after 10 min of ultrasound pretreatment
UMPSB2: Mg-laden peanut shell-based biochar after 20 min of ultrasound pretreatment
UMPSB3: Mg-laden peanut shell-based biochar after 30 min of ultrasound pretreatment
P: phosphorus
