# Supplementary material for: Characteristics and Mechanisms of Phosphorous Adsorption by Peanut Shell-Derived Biochar Modified with Magnesium Chloride by Ultrasonic-Assisted Impregnation
Source: ACS Omega. 2022 Nov 14;7(47):43102–10. doi: 10.1021/acsomega.2c05474 (PMC9713878; doi:10.1021/acsomega.2c05474)
Supplement: Supplementary file 1 — ao2c05474_si_001.pdf [file ao2c05474_si_001.pdf]

Supporting Information

Characteristics and mechanisms of phosphorous adsorption by peanut shells-derived biochar modified with magnesium chloride by ultrasonic-assisted impregnation

Xiaoqi Liu[a], Wei Zhou[a], Lei Feng[b], Lulu Wu[a], Jialong Lv[a]*, Wei Du[a]

[a] College of Natural Resources and Environment, Northwest A& F University, Yangling, Shaanxi, 712100, China

[b] College of Resource and Environment, Xinjiang Agricultural Universit, Urumqi China

***Corresponding author: Jialong Lv**

E-mail: ljlll@nwafu.edu.cn.

Postal address: Room 520, College of Natural Resources and Environment, Northwest A& F University, Yangling District, Shaanxi Province, China

Figure captions

Table S1. The C, N, and O element content of peanut shells and PSB.

Figure S1. Freundlich models (A) and Temkin model (B) for phosphate onto the biochar.

Figure S2. The effects of different initial pH values (A) and ambient temperatures (B) on the phosphate adsorption.

Table S1. The C, N, and O element content of peanut shells and PSB.

Sample	C element (%)	N element (%)	H element (%)	S element (%)
Peanut shells	47.81	0.66	5.54	0.05
PSB	77.59	1.49	2.293	0.27

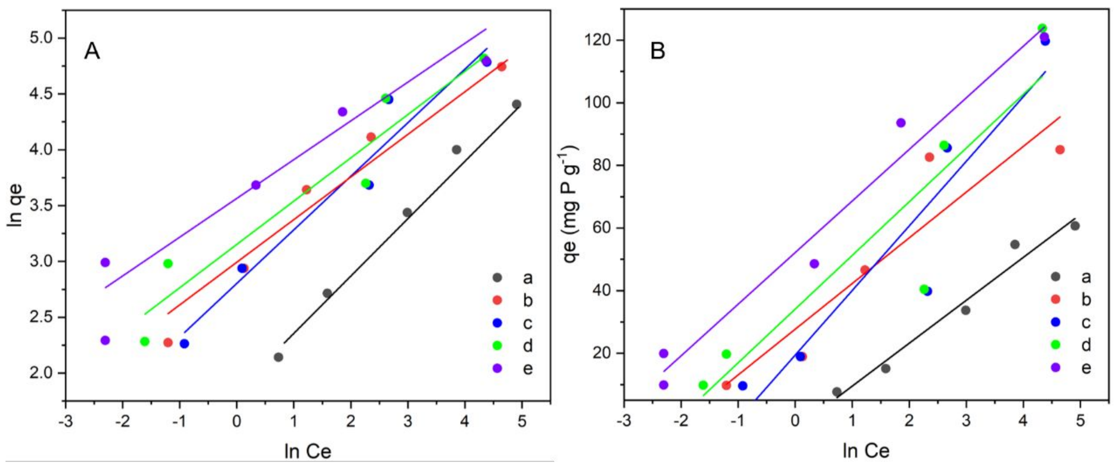

Figure S1. Freundlich models (A) and Temkin model (B) for phosphate onto the biochar.

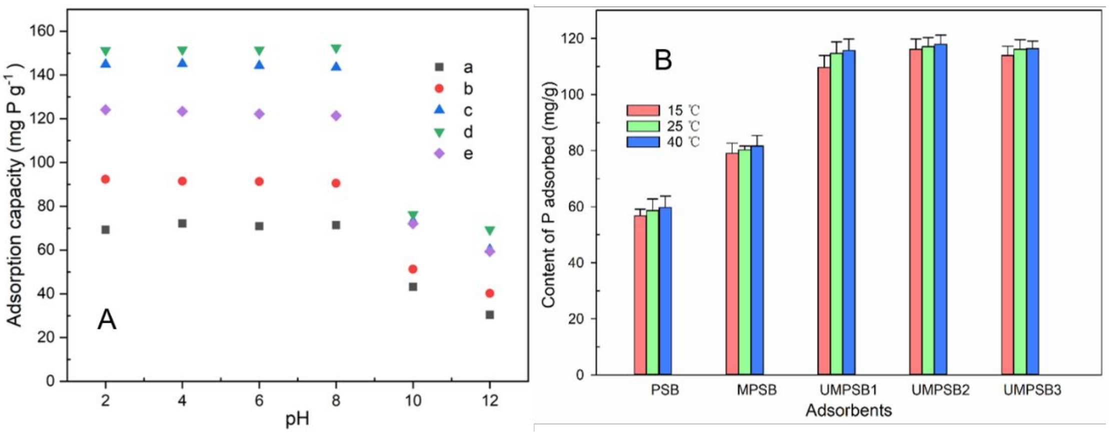

Figure S2. The effects of different initial pH values (A) and ambient temperatures (B) on the phosphate adsorption.
